# Assessing the Risk of Spreading *Clostridioides difficile* and Its Toxins Within the Dairy Farm

**DOI:** 10.3390/ani14213148

**Published:** 2024-11-02

**Authors:** Valentina A. Filippova, Larisa A. Ilina, Elena A. Yildirim, Ekaterina S. Ponomareva, Irina A. Kluchnikova, Andrey V. Dubrovin, Ksenia A. Kalitkina, Vasiliy A. Zaikin, Georgy Y. Laptev

**Affiliations:** 1 Department of Large Livestock Husbandry, St. Petersburg State Agrarian University, St. Petersburg-Pushkin 196605, Russia; deniz@biotrof.ru (E.A.Y.); dubrovin@biotrof.ru (A.V.D.); kseniya.k.a@biotrof.ru (K.A.K.); georg-laptev@rambler.ru (G.Y.L.); 2 BIOTROF LLC, St. Petersburg 196602, Russia; kate@biotrof.ru (E.S.P.); irina@biotrof.ru (I.A.K.); dfcx@biotrof.ru (V.A.Z.); 3 Faculty of Biotechnologies, ITMO University, St. Petersburg 197101, Russia

**Keywords:** silage, cattle, bacterial toxins, gut microbiome, *C. difficile*, PCR, NGS

## Abstract

*Clostridioides difficile* currently constitutes a major pathogen of the gastrointestinal tract, which poses a significant growing burden on medicine and veterinary medicine in many regions. A farm was assessed (feed table, silage pit, and feces (healthy animals, emaciated animals, and animals with mastitis)) for the presence of *C. difficile* toxins using the PCR method and for the microbiome in cow feed and feces using NGS technology, one month apart. *C. difficile* toxin A and binary toxin were detected in feed samples. *C. difficile* toxin genes were found in the feces of sick animals two to three times more often than in healthy animals. Analysis of the microbial community of cow feces revealed that, during the month, the animals experienced major changes in the community structure associated with the accumulation of pathogenic bacteria, in particular *Paeniclostridium* sp., as well as with the development of methanogenic archaea of the Methanobacteriaceae and associated microorganisms (Lachnospiraceae and Anaerovoracaceae), which may speak of a decrease in feed efficiency and, subsequently, animal productivity. Thus, it seems likely that *C. difficile* enters the gastrointestinal tract of animals through feed, while animals weakened by diseases are more sensitive to the reproduction of pathogens in the GIT due to a weakened organism.

## Simple Summary

There is currently little research on the spread and transmission of *Clostridioides difficile* within farms. A comprehensive assessment of the presence of *C*. *difficile* toxins and microbiome in cow feed and feces was conducted, which showed the presence of *C. difficile* toxin genes in silage. One month after consuming toxin-containing silage, toxin genes were detected in the feces of animals (mostly weakened), although no illnesses associated with *C. difficile* infection were reported on the farm. Given the danger of *C. difficile* to humans, it may be recommended to more carefully screen silage or animals for *C. difficile* carriage.

## 1. Introduction

The biological safety of livestock products for humans is determined primarily by the absence of pathogens and/or their toxins. *Clostridioides difficile* is a Gram-positive, endospore-forming anaerobic bacterium, often carried asymptomatically in the human gastrointestinal tract. However, when favorable conditions are met, endospores germinate in the colon, vegetative cells multiply, and toxins are produced [1], which leads to watery, non-bloody diarrhea with abdominal pain, toxic megacolon, and/or pseudomembranous colitis, which can prove fatal.

Until 2016, this bacterium was named *Clostridium difficile*. It was removed from the Clostridiaceae family and now belongs to the Peptostreptococcaceae family. *C. difficile* spores are transmitted by the fecal–oral route, and the pathogen is widespread in the environment. Recently, a large amount of information has been accumulated, indicating that infection can occur through animal products [2,3]. The researchers concluded that *C. difficile* is widespread within livestock farms. This pathogen often does not manifest itself in animals that become infection carriers [4]. *C. difficile* is often found in cattle, particularly in young animals. The prevalence was shown to be 11% in calves and 6% in adult cattle [5]. Other researchers found these bacteria in 14% [6] and 22% of calves [7], and 7% of mature animals [8].

The main virulence factors in *C. difficile* are toxin A (308 kDa) and toxin B (270 kDa), encoded by the *tcdA* and *tcdB* genes. Both constitute major clostridial toxins and are activated in response to environmental cues during late log and stationary phases. TcdA and TcdB have the same biological activity, involved in disruption of the cellular cytoskeleton, which results in cytopathic effects in cultured cells. They also have pro-inflammatory activity and can stimulate intestinal epithelial cells and immune cells to produce cytokines and chemokines [9]. Even at low doses, toxins A and B damage the dense connections of the intestinal epithelial barrier, facilitating the translocation of commensal bacteria, causing inflammation, and triggering the processes of cellular apoptosis [10]. Changes in the sequence, such as deletions and duplications at the pathogenicity locus, define the different toxinotypes of *C. difficile* [11]. Some strains may carry only one of the toxin genes (A−B+ or A+B−), yet they are reported to still cause severe diseases in humans [11]. Further on, the cytotoxicity of toxins belonging to different toxinotypes can vary, which makes the relationship between the type of strain and the severity of infection even more complex [12]. Strains lacking toxin A are reported more frequently due to deletions in those TcdA regions that can bind to receptors caused by recombination between short repetitive sequences highly conserved in this toxin gene [13]. Some strains (PCR ribotype 027 and PCR ribotype 078), especially those associated with increased morbidity and mortality, further encode a third toxin, binary toxin (or CDT) [14].

The transmission of infection from farm animals has already been confirmed by multiple studies. Significant overlap of *C. difficile* strains isolated from humans and animals has been repeatedly demonstrated, in particular for binary 5-clade toxin-positive isolates [15,16]. In the Netherlands, pigs and pig farmers were colonized with identical (no differences in single nucleotide polymorphism) and almost identical (less than two differences in SNP) clones of *C. difficile* [17]. Such a transmission cannot always be confirmed, though. For instance, in a study conducted by van Dorp and colleagues, contact with livestock was not identified as a factor in the development of community-acquired infection associated with *C. difficile* [18]. Therefore, questions pertaining to the sources and transmission routes of *C. difficile* remain open. Moreover, it should not be forgotten that both humans and animals can also be infected simultaneously from a common source (for example, from the environment).

At the same time, there is very little data on how *C. difficile* is spread within farms and in farm animal feed. Animal feed, mainly preserved, such as silage, can become a source of pathogenic bacteria and their toxins, which can cause diseases and reduce dairy cow productivity, contributing to their constant carriage and circulation inside the farm. The existing practice of applying organic fertilizers to the soil, which may potentially contain pathogenic bacteria, can contribute to the spread of infection or carrier in the case of poor-quality feed conservation. St. Petersburg is located in a risky farming zone, which often leads to problems in the feed conservation process. There is very little research on the spread of *C. difficile* in feed. This bacterium, for example, was found in hay in a study of the 70s and 80s of the last century [19]. Nonetheless, we have not found studies evaluating the spread of *C. difficile* in bulky feeds, which is especially interesting, since a violation of feed conservation processes can lead to the accumulation of *C. difficile* and subsequent transmission to animals, and, subsequently, to humans.

Thus, there are not many studies aimed at understanding the risks of the spread of *C. difficile* among cattle, especially in asymptomatic carriage. Therefore, the aim of this study is to assess the spread of *C. difficile* and its toxins on a farm, among animals and in the feed base, which will help reduce the spread of this pathogen in the environment.

## 2. Materials and Methods

### 2.1. Sampling

**Figure 1:**
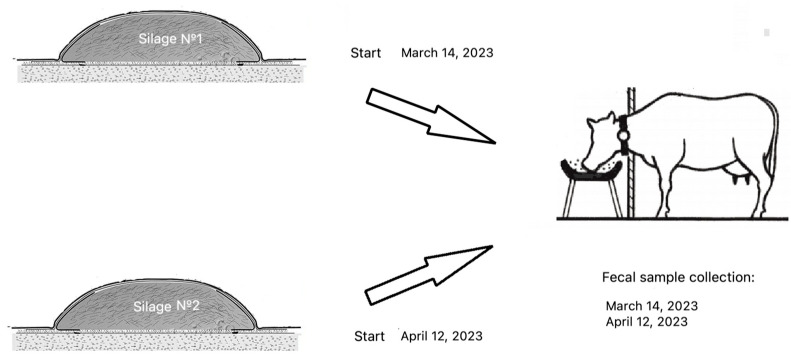
Schematic representation of the study sampling.

A comprehensive assessment of the presence of toxins and an evaluation of the microbiome in cow feed and feces was performed on a farm. The farm feed (feed table (manger), silage pit) and feces (Table 1 and Table 2, Figure 1) were studied (healthy animals, *n* = 3; emaciated animals with metabolic disorders, *n* = 3, and animals with clinical signs of mastitis, *n* = 3) to analyze the transmission and dissemination patterns of *C. difficile* toxins.

Sampling was carried out twice with a break in a month. Sampling was associated with the intake of feed from new sources (Figure 1). The months were chosen sequentially in order to assess the dynamics of accumulation and spread of toxins and/or their carriers within the farm. In March, samples of silage No. 1, feed table, and animal feces were taken; in April, samples of silage No. 2, feed table, and animal feces. Silage came from different sources, so its quality and safety were assessed. The silage consisted of a legume–cereal mixture (*Trifolium pratense* and *Phleum pratense* in equal amounts). The studied silage was laid at the end of May 2022 in 5-ton silage clamps. The silage was laid over 3 days. The average daily temperature during the silage preparation period was 11 °C. The first clamp was opened in March (the average daily temperature was 3 °C), and the second in April 2023 (the average daily temperature was 10 °C) with the start of feeding. The cows received a silage-concentrate diet adopted on the farm. The biochemical composition is presented in Table 2.

Sampling (Figure 1) was performed with the maximum possible observance of aseptic conditions with these methods. The samples were frozen (−20 °C) and transferred on dry ice to the laboratory.

### 2.2. DNA Isolation

Total DNA from silage and feces samples was isolated using the Genomic DNA Purification Kit (Thermo Fisher Scientific, Inc., Waltham, MA, USA) according to the included instructions.

### 2.3. C. difficile Toxin Analysis

Using the PCR method, the samples were examined for the presence of the genes of the binary toxin (*cdtB*), toxin A (*tcdA*), and toxin B (*tcdB*) of *C. difficile*. Amplification reactions were performed on a DT-Light amplifier (DNA Technology, Moscow, Russia). DNA fragments were visualized by gel electrophoresis in agarose gel using a tris-acetate buffer. After that, the results were photographed. The list of primers is presented in Table 3.

### 2.4. NGS Sequencing of a 16S RNA Gene Fragment

The 16S rRNA gene fragment was amplified from total DNA using the full-length primer sequences for the V3-V4 region of the 16S rRNA gene (Illumina Inc., San Diego, CA, USA). The 16S Amplicon PCR Forward Primer = 5′ TCGTCGGCAGCGTCAGATGTGTATAAGAGACAGCCTACGGGNGGCWGCAG, and the 16S Amplicon PCR Reverse Primer = 5′ GTCTCGTGGGCTCGGAGATGTGTATAAGAGACAGGACTACHVGGGTATCTAATCC. Metagenomic sequencing was carried out on a genomic sequencer MiSeq (Illumina Inc., San Diego, CA, USA) with MiSeq kit Reagent Kit v3 (Illumina Inc., San Diego, CA, USA). The maximum length of the obtained sequences was 2 × 300 nt. Chimeric sequences were excluded from analysis using the USEARCH 7.0 program (http://drive5.com/usearch/ accessed on 17 May 2023). Processing of the obtained 2 × 300 nt reads took place using the bioinformatics platform “CLC Bio GW 7.0” (Qiagen, Venlo, The Netherlands) and included overlap, quality filtering (QV > 15), and primer trimming.

Automatic bioinformatic data analysis was performed using QIIME2 ver. 2020.8 software (https://docs.qiime2.org/2020.8/ accessed on 17 May 2023). After importing the sequences in the .fastq format from the sequencing device and the creation of matching files necessary for operation (containing metadata of the studied files), paired read lines were aligned. The sequences were then filtered by quality using default settings. Noise sequences were filtered using the DADA2 package integrated into the QIIME2 software, which hosts information on the quality of sequences in its error model (filtering of chimeric sequences, artifacts, adapters), which makes the algorithm resistant to a sequence of lower quality. That being the case, the maximum length of the truncation sequence was used, equal to 250 b.p. (https://benjjneb.github.io/dada2/tutorial.html). To construct the phylogeny, multiple sequence alignments were de novo performed using the MAFFT software package (Version 7.487) (https://mafft.cbrc.jp/alignment/software/ accessed on 17 May 2023), followed by masked alignments to remove positions that differed significantly. For taxonomy assignment, the QIIME2 software was used, which assigns taxonomic identification to sequences based on ASV data (using BLAST, RDP, RTAX, mothur, and uclust methods) using the 16s rRNASilva 138.1 database (https://www.arb-silva.de/documentation/release-138.1/ accessed on 17 May 2023).

### 2.5. Statistical Data Processing

The results were mathematically and statistically processed using the method of multifactor analysis of variance (ANOVA) in Microsoft Excel XP/2003 and R-Studio (Version 1.1.453) (https://rstudio.com accessed on 17 May 2023). The reliability of the differences was determined by the Student’s *t*-test, and the differences were considered statistically significant at *p* < 0.05. Mean values were compared using Tukey’s honestly significant difference (HSD) test and the TukeyHSD function in the R StatsPackage (Version 4.5.0).

After preparing the data for principal component analysis (PCA), we performed PCA for the transposed data frame, scaling the data to unit variance, and a data set was created for plotting. This enabled performing PCA on the dataset, creating a plot of the PCA scores for the first two principal components, and coloring the points following the metadata vector. To obtain the graph, RStudio was used, in particular the ggplot2 (Version 3.5.1), vegan (Version 2.6-8), and corrplot (Version 1.2-9 )libraries.

The website https://www.interactivenn.net was used to build Venn diagrams. It is a web-based tool for the analysis of sets through Venn diagrams. It allows us to create and visualize Venn diagrams, which can be used to identify relationships and overlaps between different sets of data [21].

## 3. Results

### 3.1. Results of Assessing the Presence of Toxins Using PCR

In March, *C. difficile* toxins, genes of toxin A (*tcdA*), and binary toxin (*cdtB*), were detected in combined silage samples and samples taken from the feed table using the PCR method (Table 4). No toxin was detected in the feed samples taken in April. This is primarily due to the different sources of feed that came from different sources in March and April.

No *C. difficile* toxin was detected in fecal samples in March, while toxins were already detected in fecal samples collected in April. At the same time, genes coding for the toxin were detected more often in sick animals than in healthy ones. Thus, among the group of healthy animals, only a single animal was a carrier of the *C. difficile* binary toxin gene; other genes were not identified. Animals with clinical manifestations of mastitis were found to have all toxin genes present—*tcdA* was detected in the feces of all three animals, *tcdB* in two, and the binary toxin (*cdtB*) was detected in the feces of one animal. Toxin A and binary toxin were detected in animals with metabolic disorders in all animals studied in the group. Toxin B was not detected in any animal.

Thus, based on the data obtained, it seems feasible to introduce *C. difficile* along with feed into the gastrointestinal tract of animals, while animals weakened by diseases are more sensitive to the reproduction of pathogens in the gastrointestinal tract due to a weakened organism.

### 3.2. Results of the Taxonomic Assessment of Microbial Communities

**Figure 2:**
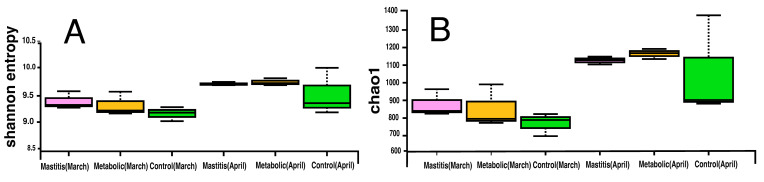
Shannon (**A**) and Chao1 (**B**) biodiversity indices of microbial communities of feces and cow feed of experimental groups in March and April.

Various indices act as measures of microbial community diversity. Based on the comparative analysis, the Shannon index, which is quite informative when used at different taxonomic levels, does not depend much on the sample size, and has good reproducibility, can be considered the most suitable for assessing the phylogenetic diversity of prokaryotes. Thus, the Shannon index for prokaryotic fecal communities in March varies between 9.1 and 9.5, and for samples taken in April—9.3–9.8 (Figure 2), and the Chao1 index varies within the range of 720–900 in March samples and 900–1150 in April ones. Generally, the samples collected in April are marked by higher biodiversity according to the Shannon index and the Chao1 index. The assessment of alpha diversity of prokaryotic communities in feces did not show any clear change in the profile of Shannon and Chao1 indices.

**Figure 3:**
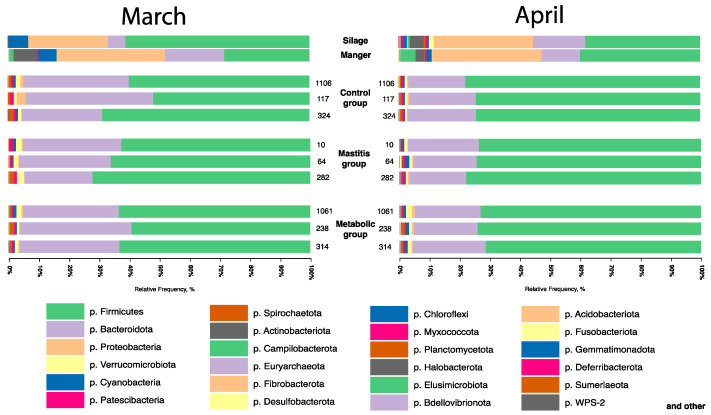
Taxonomic diversity of silage samples, feed table, and averaged feces samples of animals from experimental groups at the level of bacterial phyla.

The results of a taxonomic assessment of the microbial community of silage samples and samples from the feed table (Figure 3) showed that in March, silage samples contained 61.02% Firmicutes, 26.5% Proteobacteria 5.74%, and Bacteroidota; in April, silage samples contained 38.12% Firmicutes, 32.94% Proteobacteria, and 17.4% Bacteroidota. Samples from the feed table in March contained 28.24% Firmicutes, 35.95% Proteobacteria, and 19.6% Bacteroidota, and 39.82% Firmicutes, 36.42% Proteobacteria, and 12.61% Bacteroidota in April.

Furthermore, in the samples collected from the feed table in March, 7.82% of the presence of the phylum Actinobacteriota representatives was detected, while in April, the number of this bacteria decreased to 2.87% on the feed table. Silage samples contained 0.19% Actinobacteriota in March and 4.47% in April.

Fecal sample analysis revealed that the dominant phylum in the microbial community was the phylum Firmicutes. In March, the proportion of representatives of this phylum ranged from 60.16 ± 8.43% in healthy animals to 67.00 ± 4.81% in animals with signs of mastitis. In April, the proportion of representatives of this phylum ranged from 49.75 ± 1.38% in animals with metallic disorders to 75.55 ± 1.99% in healthy animals. The analysis revealed that, in March and April, there were no major differences in the number of this phylum between animals of different groups. Significant differences in the abundance of the phylum Firmicutes were shown between different sampling months. In the group of healthy animals, the relative number of representatives of the phylum increased by 25% (*p* = 0.027), and in animals with signs of mastitis, by 11% (*p* = 0.002) in April compared to March. The number of phyla in the group with metabolic disorders decreased by 26% (*p* = 0.046).

**Figure 4:**
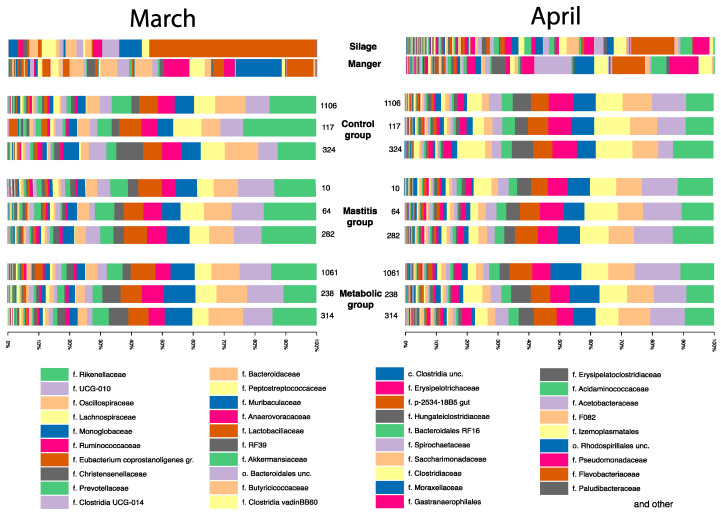
Taxonomic diversity of samples of silage, feed table, and average samples of animal feces of experimental groups at the level of bacterial families.

**Figure 5:**
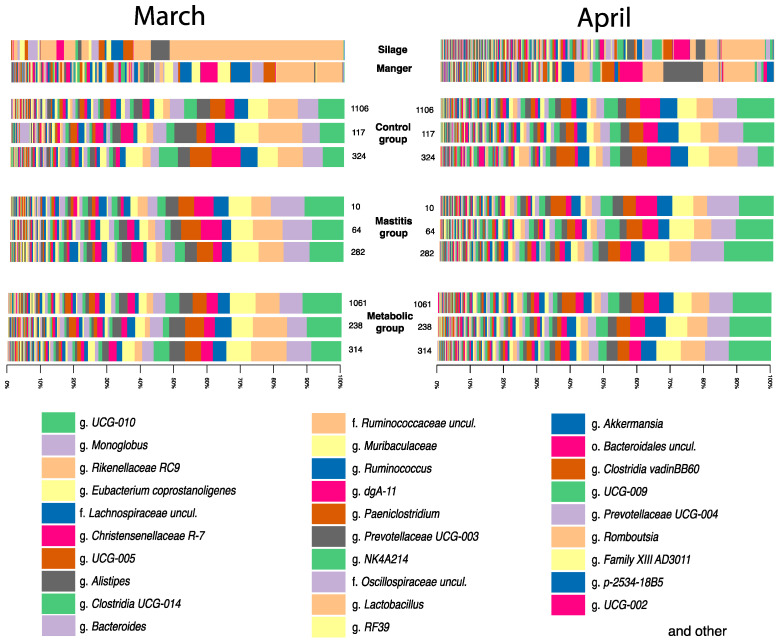
Taxonomic diversity of samples of silage, feed table, and average samples of animal feces of experimental groups at the level of bacterial genus.

**Figure 6:**
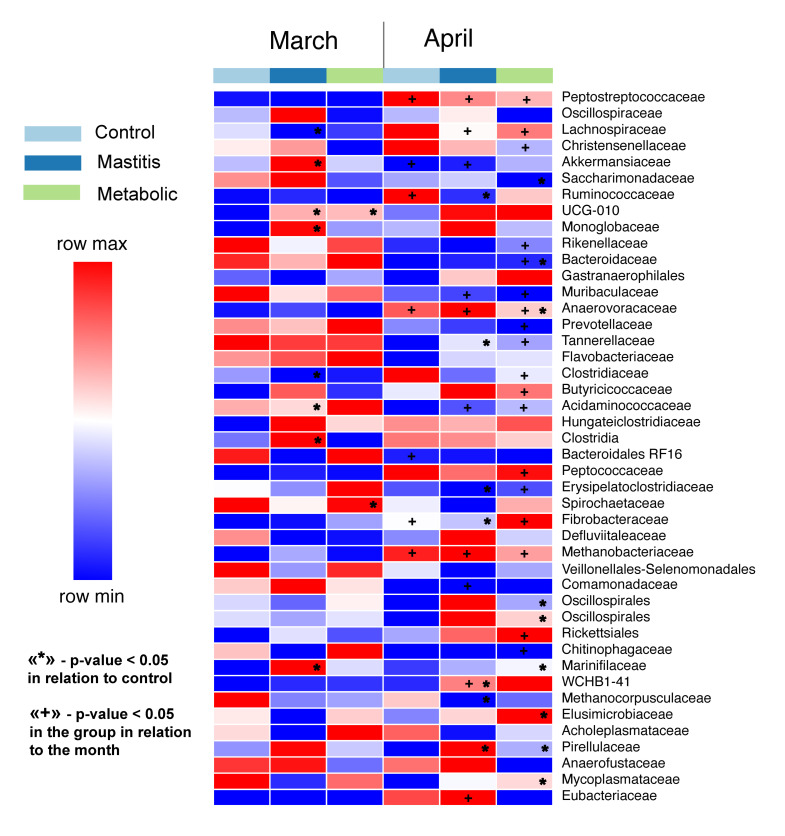
Heat map of significant differences between animal groups in April and March.

The greatest differences between the groups were observed depending on the sampling period, and significantly fewer major differences were found between the studied groups in March and April (Figure 4, Figure 5 and Figure 6). In March, minor differences were observed between animals in different groups, which were most pronounced in the differences between the group of healthy animals and the group with metabolic disorders. Thus, in the group of healthy animals, a greater number of bacteria of the Lachnospiraceae family was observed (*p* = 0.04): 7.93 ± 1.138%; while in the group of animals with mastitis, the proportion of these bacteria was 5.83 ± 0.876%.

Among the dominant bacteria in the fecal microbiota, the most numerous were bacteria of the genera *UCG-010* and *UCG-005*, belonging to the family Oscillospiraceae, as well as bacteria of the genus *Monoglobus* of the family Monoglobaceae (Figure 5). Among the dominant bacteria were *Eubacterium coprostanoligenes*, *Alistipes* sp., *Clostridia UCG-014*, *Bacteroides* sp., *Ruminococcus* sp., *Paeniclostridium* sp., *Akkermansia* sp., and others. It is interesting that the number of bacteria of the genera *UCG-010* and the genus *Monoglobus* of the family Monoglobaceae was higher in animals with diseases. The proportion of *Ruminococcus* sp., on the contrary, was higher in clinically healthy animals.

Further on, significant differences in the *UCG-010* group were revealed between sick and healthy animals in March. The number of this group was definitely higher in sick animals by 25%—7.81 ± 0.78% in the control group versus 10.53 ± 1.20% for the group with mastitis and 10.41 ± 1.39% for animals with metabolic disorders (*p* = 0.009 for animals with mastitis; *p* = 0.015 for animals with metabolic disorders). In April, no significant differences in *UCG-010* were found between the groups of sick and healthy animals. The proportion of the genus *UCG-005, Eubacterium coprostanoligenes*, *Alistipes* sp., and *Clostridia UCG-014* did not change significantly in any of the studied groups.

The number of bacteria in the family Ruminococcaceae in March was the same in all groups. In April, the number of Ruminococcaceae genus *Ruminococcus* increased by 30% in the group of healthy animals from 5.69 ± 0.693% to 7.8 ± 0.271% (*p* = 0.004), while there were no significant changes in the other groups.

From March to April, major changes occurred in the gastrointestinal tract of cows (Figure 2). The number of Anaerovoracaceae family bacteria definitely increased. In healthy animals, the proportion of this family in the community was 0.87 ± 0.066% in March, 1.05 ± 0.15 in animals with mastitis, and 0.82 ± 0.17% in animals with metabolic disorders. In April, the proportion of these bacteria significantly increased by about 2 times to 2.2 ± 0.270% (*p* = 0.0005) in healthy animals and to 2.49 ± 0.500% (*p* = 0.004) in animals with metabolic disorders, and in animals with mastitis to 1.8 ± 0.12% (*p* = 0.0004). An increase in the number of Anaerovoracaceae family bacteria was caused by a bacterium of the genus *Mogibacterium*, the proportion of which significantly increased from 0 by about 10 times, from 0.03 ± 0.02% in healthy animals to 0.2 ± 0.11% (*p* = 0.003), from 0.02 ± 0.02% to 0.25 ± 0.08% in animals with mastitis (*p* = 0.004), and from 0.01 ± 0.01% to 0.12 ± 0.03% (*p* = 0.003).

From March to April, changes in the gastrointestinal tract of cows were definitely associated with the number of bacteria of the family Peptostreptococcaceae *Paeniclostridium* sp. Its relative abundance in April samples increased several times compared to March. In healthy animals, it increased from 0.15 ± 0.041% to 3.82 ± 1.620% (*p* = 0.03). In animals with mastitis, from 0.09 ± 0.076% to 2.9 ± 1.619% (*p* = 0.02). In animals with metabolic disorders, from 0.09 ± 0.082% to 2.45 ± 1.496% (*p* = 0.03).

These bacteria were only detected in the fecal samples of the cows under study. They were not detected in the silage and in the feed table.

The number of methanogenic archaea of the Methanobacteriaceae family also increased during this period. From 0 values in the March samples, the proportion of Methanobacteriaceae increased 5–10 times in all animal groups to 0.22 ± 0.070% (*p* = 0.01) in the group of healthy animals, to 0.24 ± 0.028% (*p* = 0.04) in the group of animals with mastitis, and to 0.17 ± 0.071% (*p* = 0.03) in the group of animals with metabolic disorders.

From March to April, the number of bacteria of the genus *Cellulosilyticum* and the NK3A20 group of the Lachnospiraceae family increased significantly. In March, NK3A20 was not detected in any sample. In April, the relative number in the group of healthy animals was 7.8 ± 0.266% (*p* = 0.00003), in the group of animals with mastitis it was 6.9 ± 0.061% (*p* = 0.000007), and in the group of animals with metabolic disorders it was 6.6 ± 0.134% (*p* = 0.0003). The number of *Cellulosilyticum* sp. was also insignificant in the March samples, but by April it definitely increased by more than an order of magnitude (*p* = 0.0007 for the control group; *p* = 0.000004 for animals with mastitis; *p* = 0.0007 for animals with metabolic disorders).

**Figure 7:**
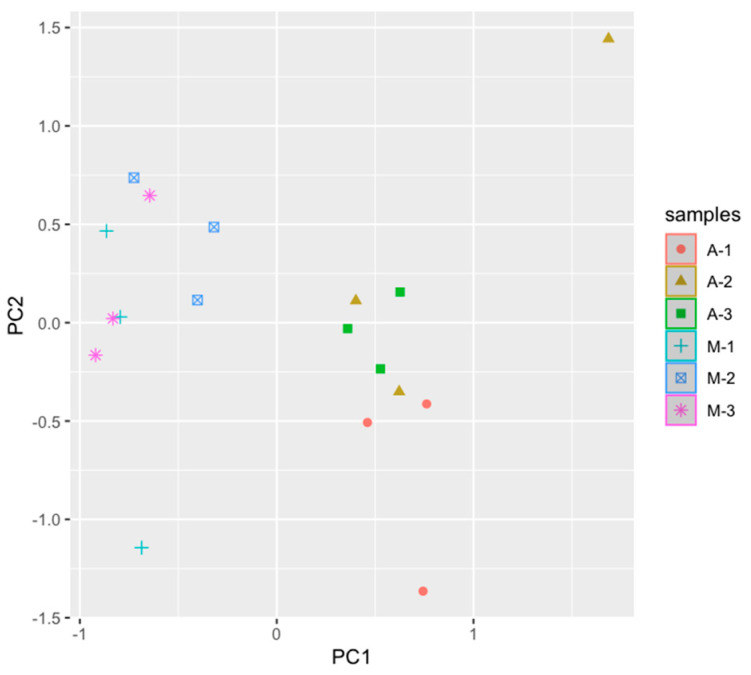
The principal coordinate analysis of cattle fecal microbiota. A—April, M—March, 1—control group, 2—group of animals with mastitis, 3—group of animals with metabolic disorders.

The analysis of the main components revealed the identification of two generalized groups of samples (Figure 7) related to the sampling time. This, therefore, confirms the major changes that occurred during this period, which may be directly related to the ingestion of *C. difficile* from feed into the gastrointestinal tract of animals.

**Figure 8:**
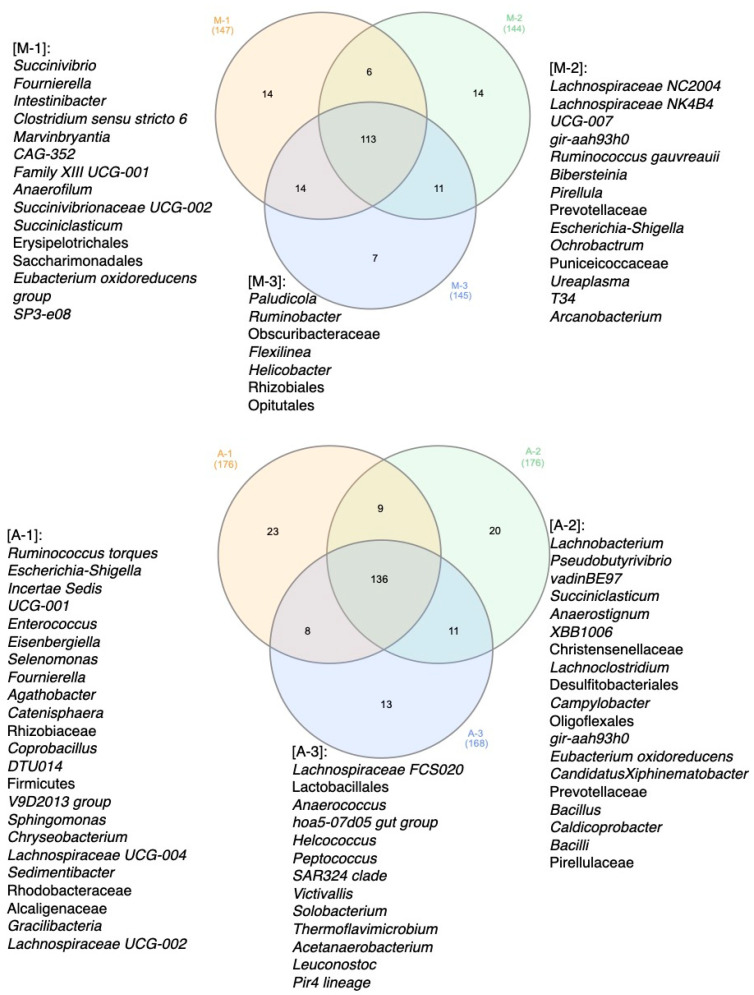
Venn diagrams of differences in the cattle fecal microbiota microbiome, reflecting the unique microorganisms in each group of experienced cows. A—April, M—March, 1—control group, 2—group of animals with mastitis, 3—group of animals with metabolic disorders.

The microbial community biodiversity of cow feces was also assessed using Venn diagram analysis at the bacterial genera level (Figure 8). Some bacterial genera were unique to certain groups, with about 113 genera common to all groups in March and 136 in April. In March, however, there were fewer unique births for each group than in April. Thus, in the group of healthy animals, the number of unique genera increased from 14 to 23, in the group with mastitis from 14 to 20, and in the group with metabolic disorders from 7 to 13. Generally, the group of healthy animals was distinguished by a wide variety of unique bacteria.

## 4. Discussion

*Clostridioides* (*Clostridium*) *difficile* currently constitutes a major pathogen of the gastrointestinal tract, which creates a significant growing burden on medicine and veterinary medicine in many regions of the world [22]. Human *C. difficile*-associated infections have become more common in the community [22] and appear to be linked to animal and environmental sources of *C. difficile* [23]. There are a number of studies showing the coincidence of *C. difficile* strains isolated from humans and animals [24,25,26,27]; however, zoonotic transmission of *C. difficile* has never been definitively demonstrated, and the process of spreading the bacterium inside farms has been extremely poorly studied.

In our study, we found genes coding *C. difficile* toxins in the feed and gastrointestinal tract of the animals studied. However, we were unable to detect even the *Clostridioides* genus based on the results of sequencing the V3–V4 region of the 16S rRNA gene fragment of the gastrointestinal microbiome. Analysis of the V3–V4 region allows for an assessment of microbiomes at the genus level, as sequencing of the V3–V4 region limits analysis to the genus level, and species differentiation often cannot be achieved [28]. The absence of *C. difficile* in the studied samples may be due to difficulties in extracting DNA from endospores. As a rule, the isolation method on nutrient media or using PCR is used to detect *C. difficile* [29]. Detection using targeted sequencing is likely to be ineffective due to the small amounts of pathogen involved, particularly in the absence of prominent associated clinical manifestations.

To date, very little is known about the distribution of *C. difficile* in dairy cattle. Studies conducted in Europe and Canada have shown that the prevalence of *C. difficile* ranges from 0.95 to 10% in adult dairy cows [30] and from 6 to 56% [31] in calves. In the dairy industry, especially where small- and medium-sized farms predominate, farm workers are more likely to come into contact with animals than on beef farms. Our study demonstrated the dynamics of the presence of *C. difficile* toxin genes depending on the time of sampling and the possible transfer of pathogens through feed. In the March samples, the genes of toxin A (*tcdA*) and binary toxin (*cdtB*) were detected only in samples from the feed table and silage, while they were not detected in fecal samples. A month later, in samples collected in April, the genes of toxins A (*tcdA*) and B (*tcdB*) and binary toxin (*cdtB*) (Table 4) were detected in varying quantities in the feces depending on the group. Interestingly enough, the highest number of toxin genes was found in groups of sick cows compared to the control group. The accumulation of toxin genes in these groups can be explained by a reduced adaptive capacity in sick animals, which allowed *C. difficile* to multiply more effectively in the gastrointestinal tract of a weakened animal.

Significant changes occurred in the gastrointestinal tract microbiome of cows from March to April. The number of methanogenic archaea of the family Methanobacteriaceae and bacteria of the family Anaerovoracaceae *Mogibacterium* sp. increased. Hegarty et al. [32] indicated a negative relationship between feed efficiency and methane emissions in beef cattle. In vivo studies have also revealed that inhibition of methanogens reduces the acetate:propionate ratio, reflecting a shift in fermentation towards more reduced volatile fatty acids than towards acetate [33,34]. About 2–12% of the feed energy consumed is also lost in the form of methane [35]. Some studies have noted a relationship between methane release and *Mogibacterium* sp. in cows. Thus, Smith et al. [36], having ranked animals by the level of residual methane emissions, found that the number of bacteria of the genus *Mogibacterium* was significantly increased in animals with a high level of methane emissions. In addition, the abundance of *Butyrivibrio* sp. and *Mogibacterium* sp. was negatively related to the propionate percentage. That being the case, changes associated with the accumulation of toxin-producing bacteria negatively affected the state of the gastrointestinal microbiome of all groups of animals studied, which in this case turn out to be carriers, without clinical manifestations of *C. difficile* infection.

Most rumen methanogens use hydrogen gas as a substrate for methanogenesis, which is produced as a result of carbohydrate fermentation by rumen bacteria, protozoa, and fungi [37]. The Lachnospiraceae family is widespread in the intestinal environment and forms part of the core rumen microbiome. Lachnospiraceae NK3A20 is assumed to possess hydrogenases [38]. From March to April, the number of bacteria of the genus *Cellulosilyticum* and the NK3A20 group of the Lachnospiraceae family increased significantly. The abundance of *Cellulosilyticum* sp. was also insignificant in the March samples, but by April it had significantly increased by more than an order of magnitude.

From March to April, changes in the gastrointestinal tract of cows were reliably associated with the number of pathogenic Peptostreptococcaceae bacteria, *Paeniclostridium* sp. Its relative abundance in April samples increased several times compared to March. *Paeniclostridium sordellii* is a Gram-positive anaerobic bacterium that conditionally pathogenically causes acute infectious diseases in humans and animals, including myonecrosis, enterotoxemia, sepsis, and toxic shock [39]. *Paeniclostridium sordellii* synthesizes two homologous exotoxins: lethal toxin (TcsL) and hemorrhagic toxin (TcsH) [40]. These belong to the large clostridial toxin (LCT) family, which also includes *Clostridioides difficile* toxin A (TcdA) and toxin B (TcdB), *Clostridium perfringens* large cytotoxin (TpeL), and *Clostridium novyi* alpha toxin (Tcnα). This bacterium was not detected in the feed and feed table (manger) samples either in March or April, so it was apparently not introduced from an external source, but was a permanent inhabitant of the gastrointestinal tract of cows. They may be constantly present on the farm, circulating through the gastrointestinal tract–manure–feed system without causing any obvious clinical manifestations.

The conducted principal coordinate analysis demonstrated the identification of two generalized groups of samples associated with the time of sampling. This, therefore, confirms the major changes that occurred during this period, which may be directly related to the ingestion of *C. difficile* from feed into the gastrointestinal tract of animals. Apparently, in this case, we can talk about the carrier of *C. difficile* in the studied animals, since no clinical signs of the development of clostridiosis were noted during this period. At the same time, a greater number of genetic determinants of toxins were identified in the feces of sick and weakened animals. This can lead to increased risks of *C. difficile* transmission further down the food chain to humans.

## 5. Conclusions

A comprehensive assessment of the presence of C*. difficile* toxins and an assessment of the microbiome in the feed and feces of cows was performed. As a result, accumulation of genetic determinants of *C. difficile* toxins (toxin A (*tcdA*), toxin B (*tcdB*), and binary toxin (*cdtB*)) entering the gastrointestinal tract from feed was shown in all studied groups of cows. A greater number of genetic determinants of toxins was detected in the feces of sick and weakened animals, compared to the control group of healthy animals. No clinical manifestations of the development of *C. difficile* infection in animals were observed, which suggests asymptomatic pathogen carriage. At the same time, the analysis of the microbial community of cow feces revealed that, during the month, the animals had significant changes in the structure of the gastrointestinal tract community associated with the accumulation of pathogenic bacteria, in particular *Paeniclostridium sordellii*, as well as with the development of methanogenic archaea of the Methanobacteriaceae family and related microorganisms (Lachnospiraceae and Anaerovoracaceae (*Mogibacterium* sp.)), which may speak of a decrease in feed efficiency and, subsequently, animal productivity. Therefore, despite the absence of signs of diseases associated with *C. difficile* infection, weakened animals can serve as a source for the transmission and spread of this pathogen in the environment and be a source of danger to humans.

## Figures and Tables

**Table 1:** List of animals by group.

Cow	Name	Group	Calving Date
No. 10	Soiuznitsa	Mastitis	13 June 2022
No. 64	Laura	Mastitis	27 July 2022
No. 282	Delfinka	Mastitis	19 September 2022
No. 314	Ovatsiia	Metabolic disorders ^1^ (exhaustion, diarrhea)	21 October 2022
No. 238	Tara	Metabolic disorders (emaciation)	10 October 2022
No. 1061	Sultanka	Metabolic disorders (emaciation, joint damage)	18 May 2022
No. 324	Alaska	Clinically healthy (control)	15 January 2022
No. 1106	Obaiatelnaia	Normal (control)	30 January 2022
No. 117	Lorena	Normal (control)	2 February 2022

^1^ Metabolic disorders of unknown etiology.

**Table 2:** Biochemical characteristics of silage samples.

Indicator	Unit	Silage No. 1	Silage No. 2
Feeding units	FU/kg	0.25	0.21
Lactic acid	%	3.99	3.86
Acetic acid	%	0.58	0.57
Butyric acid	%	0.01	0.35
Mass fraction of moisture	%	63.2 ± 0.3	70.0 ± 0.5
Mass fraction of dry matter	%	36.8 ± 1.9	30 ± 1.8
Mass fraction of crude protein	%	3.06 ± 0.4	3.08 ± 0.4
Mass fraction of crude fat	%	0.99 ± 0.42	0.81 ± 0.1
Mass fraction of crude ash	%	2.2 ± 0.1	2.2 ± 0.1
Mass fraction of crude fiber	%	11.3 ± 1.5	8.8 ± 1.4
Exchange energy	MJ/kg	3.40	2.78
Active acidity	pH units	3.7 ± 0.1	3.7 ± 0.1
Mass fraction of soluble carbohydrates	%	1.1 ± 0.3	0.1 ± 0.01

**Table 3:** Sequences of primers used in this study.

Primer	Sequences (5′–3′)	b.p.	Refference
*t* *cdA*	GCATGATAAGGCAACTTCAGTGGTA AGTTCCTCCTGCTCCATCAAATG	629	[20]
*tcdB*	CCAAARTGGAGTGTTACAAACAGGTG GCATTTCTCCATTCTCAGCAAAGTA	410	[20]
*c* *tdB*	TTGACCCAAAGTTGATGTCTGATTG CGGATCTCTTGCTTCAGTCTTTATAG	262	[20]

**Table 4:** Distribution of the proportion of detected *C. difficile* toxins in fecal and feed samples in March and April.

	March			April		
	*cdtB*	*tcdA*	*tcdB*	*cdtB*	*tcdA*	*tcdB*
Mastitis	1/3 *	3/3	2/3	1/3	3/3	2/3
Metabolic	3/3	3/3	0/3	3/3	3/3	0/3
Healthy	1/3	0/3	0/3	1/3	0/3	0/3
Silage	1/1	1/1	0/1	0/1	0/1	0/1
Manger	1/1	1/1	0/1	0/1	0/1	0/1

* The ratio of the proportion of positive samples to the total number of samples.

## Data Availability

The 16S rRNA gene sequences determined in this study were deposited in the NCBI Sequence Read Archive (SRA) database (SRA accession numbers SAMN43079369—SAMN43079379).
